# chi-miR-324-3p Regulates Goat Granulosa Cell Proliferation by Targeting *DENND1A*

**DOI:** 10.3389/fvets.2021.732440

**Published:** 2021-11-18

**Authors:** Yufang Liu, Yulin Chen, Zuyang Zhou, Xiaoyun He, Lin Tao, Yanting Jiang, Rong Lan, Qionghua Hong, Mingxing Chu

**Affiliations:** ^1^Key Laboratory of Animal Genetics, Breeding and Reproduction of Ministry of Agriculture and Rural Affairs, Institute of Animal Science, Chinese Academy of Agricultural Sciences, Beijing, China; ^2^College of Life Sciences and Food Engineering, Hebei University of Engineering, Handan, China; ^3^Yunnan Animal Science and Veterinary Institute, Kunming, China

**Keywords:** chi-miR-324-3p, *DENND1A*, granulosa cells, proliferation, goat

## Abstract

Granulosa cell (GC) proliferation provides essential conditions for ovulation in animals. A previous study showed that *DENND1A* plays a significant role in polycystic ovary syndrome. However, the modulation of *DENND1A* in GCs remains unclear. Our previous integrated analysis of miRNA–mRNA revealed that the 3'-untranslated region of *DENND1A* could be a target of chi-miR-324-3p. In this study, we used quantitative reverse transcription polymerase chain reaction (RT-qPCR) to investigate *DENND1A* expression in ovarian tissues of high- and low-yielding goats. Furthermore, dual-fluorescent reporter vector experiments, Cell Counting Kit-8 (CCK-8) assay, and RT-qPCR were used to elucidate the regulatory pathway of chi-miR-324-3p-*DENND1A* in GCs. The results revealed an opposite tendency between the expressions of chi-miR-324-3p and *DENND1A* in the ovaries of high- and low-yielding goats. The CCK-8 assay indicated that chi-miR-324-3p overexpression significantly suppressed GC proliferation, whereas chi-miR-324-3p inhibition promoted GC proliferation. In addition, the expressions of GC proliferation markers *LHR, Cylin D2*, and *CDK4* showed the same tendency. The dual-fluorescent reporter assay revealed that chi-miR-324-3p directly targeted *DENND1A*, and the RT-qPCR results revealed that *DENND1A* expression was inhibited by chi-miR-324-3p. In summary, chi-miR-324-3p inhibited the proliferation of GCs by targeting *DENND1A*.

## Introduction

Ovaries, which have two functions, the cyclical production of fertilizable ova and steroid hormones, are important organs for economic animals (1). Follicles are the fundamental unit of mammalian ovaries, and the complex regulation of follicular dynamics dictates the degree of prolificacy in different species (2). The process of physical contact between granulosa cells (GCs) and oocytes can aid in oocyte growth, maturation, and, ultimately, ovulation. The proliferation of GCs is elementary for follicle and oocyte development, ovulation, and luteinization (3). Many factors can promote the proliferation of GCs, including genes, transcription factors, non-coding RNAs, and hormones. *BMPR-IB*, a gene that is well-known for its mutation in prolific sheep, was knocked out using the CRISPR-Cas technology in goat GCs, which resulted in the higher expressions of *R-Smads, LHR*, and *FSHR* in the *BMPR-IB* knocked out GCs compared with those in the GCs in which *BMPR-IB* was not knocked out (4). Other genes were also found to play an important role in GC development, such as *GDF9, BMP15*, and *YAP1* (5–7).

Studies have shown that miRNA as a regulator influences the development of GCs. miR-224 is a promising marker of polycystic ovary syndrome (PCOS), and the overexpression of miR-224 reduces cell expansion and expression of oocyte development-associated genes (8). miR-101-3p mimics and negative control were transfected into goat GCs, and RNA-seq was used to construct the cDNA libraries. Studies have suggested that miR-101-3p targeted to *STC1* regulates the expression of *STAR, CYP19A1, CYP11A1*, and 3β-HSD steroid hormone synthesis-associated gene, thereby promoting the secretion of E_2_ and P_4_ (3). Additionally, miR-383 and miR-320 regulate steroid hormone secretion in GCs, whereas miR-21-5p, miR-503, miR-424, and miR-503 are involved in ovulation, follicular-luteal transformation, and luteinization (9–11). miR-324-3p is involved in various cancer development processes, which promote tumor growth by targeting *DACT1* and activating the Wnt/β-catenin pathway in hepatocellular carcinoma (12). Recently, a new study demonstrated that lncRNA H1FX-AS1 targeted *DACT1* and inhibited cervical cancer by sponging miR-324-3p (13). Fang et al. revealed that lncRNA SNHG22 facilitated malignant phenotypes in triple-negative breast cancer by sponging miR-324-3p and upregulating *SUDS3* (14). However, the function of miR-324-3p in regulating GC proliferation remains unknown.

*DENND1A*, which encodes for a clathrin-binding protein that is a member of the connecdenn family and a guanine nucleotide-exchange factor, is located at 9q22.32 (15). A previous study revealed a *DENND1A* variant, DENND1A.V2, which was upregulated in the theca cells of women with PCOS, and reduced *CYP17A1* expression and androgen secretion (16). Several studies have found that polymorphisms of *DENND1A* were related to an increased risk of PCOS (17, 18). Therefore, the previous studies showed that *DENND1A* plays a crucial role in PCOS. However, the function of *DENND1A* in GCs is still unknown. In our previous study, integrated analysis of miRNA–mRNA results revealed that *DENND1A* is a target gene of chi-miR-324-3p. Hence, we deduced that chi-miR-324-3p inhibits GC proliferation in PCOS by suppressing *DENND1A* expression.

In this study, we aimed to identify the roles of chi-miR-324-3p in the proliferation of goat GCs and the relationship of chi-miR-324-3p with *DENND1A*. The findings demonstrated that chi-miR-324-3p could regulate GC proliferation by targeting *DENND1A*. Overall, this study provides new evidence supporting the potential application of chi-miR-324-3p to prolific goats.

## Materials and Methods

### Ethics Statement

This study and all the experimental procedures were approved by the Science Research Department (in charge of animal welfare issue) of the Institute of Animal Sciences and Chinese Academy of Agricultural Sciences (IAS-CAAS) (Beijing, China). Ethical approval was also provided by the animal ethics committee of IAS-CAAS (No. IAS2019-63).

### Animal Tissue Collection

Female native domestic goats, known as Yunshang black goats, were used in this study. According to their yield records (at least two-born litter size), 277 female goats (152 high yielding goat and 125 low yielding goat) with no significant differences in weight and height were grouped into either the high-yielding group (average litter size 3.00 ± 0.38) or low-yielding group (average litter size 1.32 ± 0.19) (*p* < 0.05). All goats were bred under the same conditions, with free access to water and feed in a goat farm located in Yunnan Province. In the follicular phase, three high-yielding (high litter size group, HF) and three low-yielding goats (low litter size group, LF) were selected from the batch of goats according to their litter size. The ovarian tissues were collected from goats, frozen immediately in liquid nitrogen, and subsequently stored at −80°C until RNA extraction.

### Cell Culture and Transfection

Following previously described methods, primary goat GCs were isolated from the follicles of ovarian tissues in the follicular phase (19). The diameter of the GCs in the follicular phase was over 3.5 mm. The isolated cells were seeded in 6-mm plates and maintained in a complete medium [DMEM/F12 (1:1), 10% FBS, and 1% penicillin/streptomycin] as described by Yang et al. (20). When the cell confluence was >90%, the cells were transferred to 10-mm plates for the next experiment.

### RNA Extraction

According to the manufacturer's protocol, total RNA was isolated from ground ovarian tissue powder and goat primary GCs using TRIzol reagent (Invitrogen, Carlsbad, CA, USA). The purity and concentration of the RNA samples were evaluated using a NanoDrop 2000 spectrophotometer (Thermo Scientific, Wilmington, DE, USA). Standard denaturing agarose gel electrophoresis was used to determine the presence of contamination and degradation.

### Vector Construction

The 3′ untranslated region (UTR) of *DENND1A* containing the predicted target site, including wild type (WT) and mutant type (MUT), were cloned into pmiR-RB-Report vector [named pmiR-RB-DENND1A-WT and pmiR-RB-DENND1A-MUT; isolated using *Xho*I and *Not*I (Takara, Dalian, China)], respectively. The insert sequence was synthesized by RiboBio company, including the wild/mutant sequences, miRNA mimic, mimic-NC, inhibitor, and inhibitor NC (RiboBio, Guangzhou, China).

### Dual-Luciferase Reporter Analysis

HEK293T, a human cell line of renal epithelial cells, was used to validate the miRNA target. Cells were seeded into 24-well plates. Cotransfection with 200 ng of target mRNA-3'-UTR-WT or target mRNA-3'-UTR-MUT and 10 μl of miRNA mimic or mimic-NC was performed using Lipofectamine 2000 (Invitrogen, USA). Subsequently, luciferase activity was measured using the Dual-Luciferase Reporter Assay System (Promega, WI, USA) at 48 h post-Transfection. The assays were performed in triplicate.

### Real-Time Polymerase Chain Reaction Assay

According to the manufacturer's instructions, reverse transcription was performed using a PrimeScript™ RT reagent kit (TaKaRa, Dalian, China), for mRNA and miRcute Plus miRNA First-Strand cDNA kit (TIANGEN, Beijing, China) for miRNA. RT-qPCR was performed using SYBR Green qPCR Mix (TaKaRa, Dalian, China) for mRNAs and miRcute Plus miRNA qPCR kit (TIANGEN, Beijing, China) for miRNAs using RocheLight Cycler^®^480 II system (Roche Applied Science, Mannheim, Germany). The qPCR for mRNAs was performed as follows: initial denaturation at 95°C for 5 min, followed by 40 cycles of denaturation at 95°C for 5 s, and finally annealing at 60°C for 30 s. In contrast, the qPCR for miRNAs was performed as follows: initial denaturation at 95°C for 15 min, followed by 40 cycles of denaturation at 94°C for 20 s, and finally annealing at 60°C for 34 s. The data were analyzed using the 2^−ΔΔCt^ method. *LHR, Cylin D2*, and *CDK4* were selected as GC proliferation markers (21). Goat *RPL19* gene and U6 gene were used as a reference gene for normalizing target gene data. The sequences of RT-qPCR primers are listed in Table 1.

**Table 1:** Primer sequences used for quantitative-polymerase chain reaction in this study.

**Gene name**	**GenBank accession number**	**Primer sequence 5′-3′**	**Product size (bp)**
*DENND1A*	XM_018055747.1	F: AACGCTCTGAAAATCGAGCC	228
		R: CAGCTTATCACTCCCGGCAT	
*LHR*	NM_001314279.1	F: CGGCTGGCTTTTTCACTGTT	226
		R: GGGAGGCAAATGCTGACCTT	
*Cylin D2*	XM_005680985.3	F: ATGTGGATTGCCTCAAAGCC	152
		R: CAGGTCGATATCCCGAACATC	
*CDK4*	XM_005680266.3	F: GAGCATCCCAATGTTGTCAGG	172
		R: ACTGGCGCATCAGATCCTTT	
*RPL19*	XM_005693740.3	F: ATCGCCAATGCCAACTC	154
		R: CCTTTCGCTTACCTATACC	
chi-miR-		F: CCCCAGGTGCTGCT	
324-3p			
U6		F: CAAGGATGACACGCAAATTCG	

### Ethynyldeoxyuridine Incorporation Assay

GC proliferation was quantified using an ethynyldeoxyuridine (EdU) kit according to the manufacturer's protocol (Beyotime). Cells were seeded into 96-well plates as described above. One hundred microliters of 50 μM EdU was aliquoted to each well and cells were incubated an additional 3 h. Cells were then washed with PBS and fixed with 4% paraformaldehyde for 30 min. To neutralize excess aldehyde groups, 50 μl of 2 mg/ml of glycine was aliquoted per well and incubated with cells for 15 min. Subsequently, 100 μl of 0.5% Triton X-100 in PBS was aliquoted per well and incubated with cells for 15 min. After rinsing, 100 μl of Apollo reagent was added, and the cells were incubated in the dark for 30 min at room temperature. Cells were washed with PBS and then nuclei were stained with Hoechst 33,342 reaction solution for 30 min in the dark. EdU-stained cells were visualized and quantified using a fluorescence microscope. Three fields were randomly selected for quantification and statistical analysis.

### Cell Counting Kit-8 Assay of GCs

GCs were inoculated into 96-well plates with approximately 100 μl of cell suspension per well in three replicates. Cells were then incubated for 2–4 h at 37°C in an incubator, and used for subsequent experiments after cell apposition/adequate confluency was reached more than 90%. Cell proliferation assay was conducted using the Cell Counting Kit-8 (CCK-8) method (Beyotime, Beijing, China). Goat GCs were cultured in 96-well plates and measured by adding 10 μl of CCK-8 solution per well as per protocol. After the addition of CCK-8 solution, absorbance at 450 nm was measured at 0, 6, 12, 24, 48, and 72 h.

### Statistical Analysis

Statistical analyses of RT-qPCR results and graphs were conducted using GraphPad Prism (v.5.0) (San, Diego, CA, USA). Statistical significance of the data was tested using paired *t*-tests. The results were presented as means ± SEM of three replicates, and the statistical significance was represented by *p*-values < 0.05 (*p < 0.05) and *p*-values < 0.01 (**p < 0.01).

## Results

### Expressions of chi-MiR-324-3p and DENND1A in Ovarian Tissues

**Figure 1:**
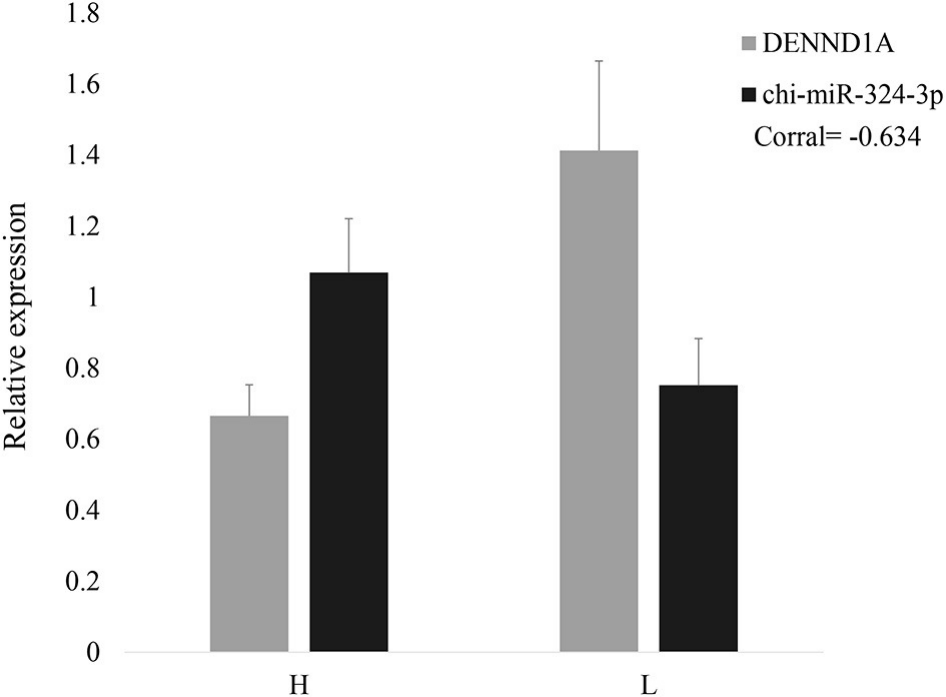
Relative expressions of *DENND1A* and chi-miR-324-3p in goat ovarian tissues in high- and low-yielding groups. H represents the high-yielding group; L represents the low-yielding group.

It has been predicted in a previous study that as a *DENND1A* regulator, chi-miR-324-3p is expressed in goat ovarian tissues in the follicular phase. To illustrate the correlation of the predicted miRNA–mRNA pair, the expression patterns of chi-miR-324-3p and its putative target *DENND1A* were investigated in HF (high-yielding group) and LF (low-yielding group). As shown in Figure 1, opposite expression patterns of chi-miR-324-3p and *DENND1A* were observed in the high-yielding group (Figure 1). This indicated a negative correlation between the expressions of chi-miR-324-3p and *DENND1A*.

### Overexpression of chi-MiR-324-3p Suppressed GC Proliferation

**Figure 2:**
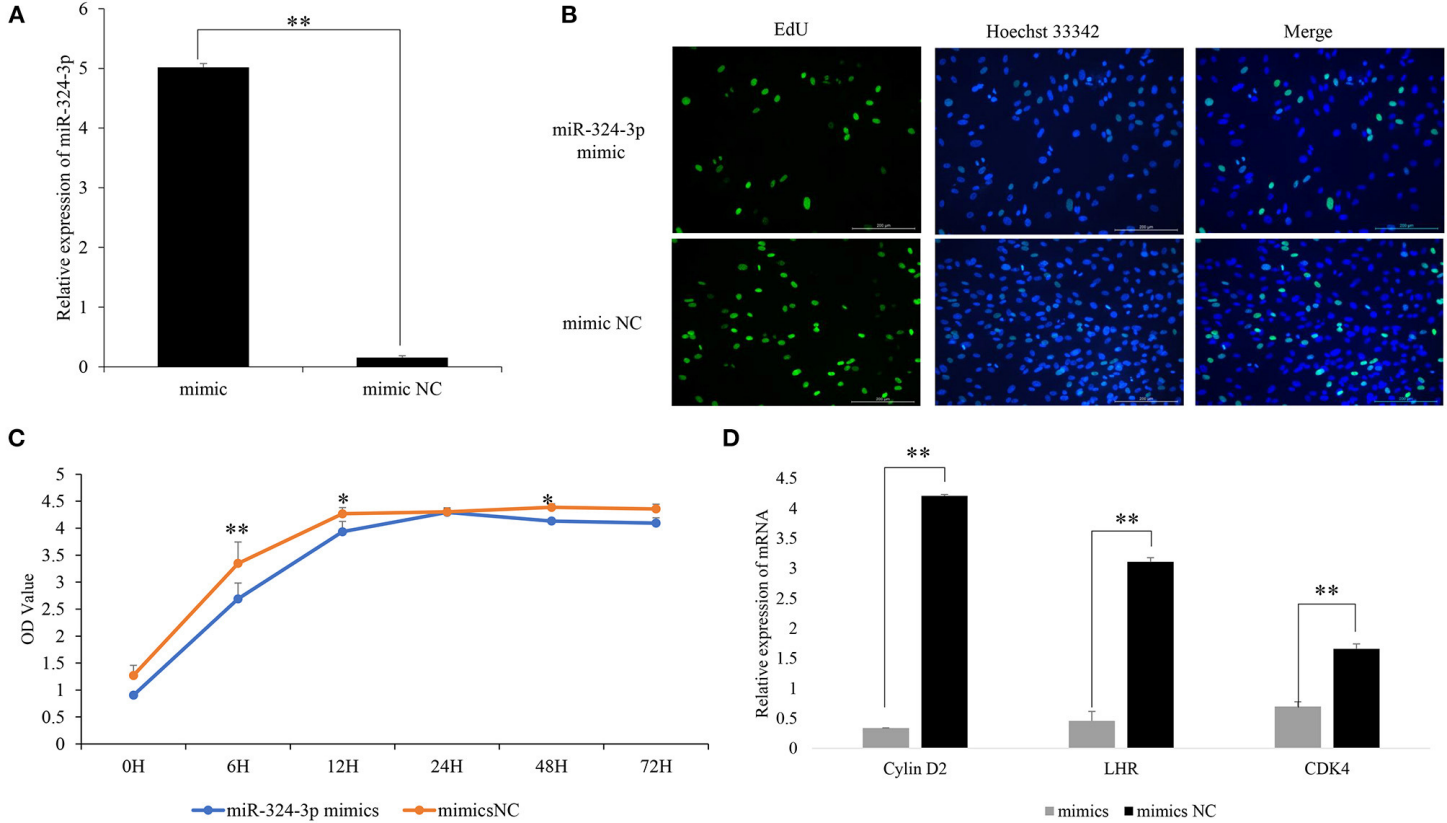
Overexpression of chi-miR-324-3p in goat GCs. **(A)** Relative expression of miR-324-3p. **(B)** The result of EdU assay. **(C)** CCK-8 assay of goat GCs with miR-324-3p mimic and mimic NC transfection at 0–72 h. **(D)** Relative expressions of *LHR, CDK4*, and *Cylin D2* in transfected chi-miR-324-3p mimic and mimic NC at 48 h. *p < 0.05; **p < 0.01. CCK-8, Cell Counting Kit-8; GC, granulosa cell.

To investigate the regulatory function of chi-miR-324-3p on GC proliferation, an overexpression experiment was performed by transfecting a chi-miR-324-3p mimic into goat GCs (Figure 2A). The EdU, CCK-8 assay and analysis of the expressions of GC proliferation factors were used for validating the functions of chi-miR-324-3p in GCs. The results revealed that the GCs proliferation was significantly lower in the mimic group than that in the mimic-NC group (Figures 2B,C) (*p* < 0.05). The expressions of *LHR, Cylin D2*, and *CDK4* were also significantly lower in the mimic group than in the mimic-NC group (Figure 2D) (*p* < 0.05). These results demonstrated that the overexpression of chi-miR-324-3p inhibited the proliferation of goat GCs.

### Inhibition of chi-MiR-324-3p Promoted the Proliferation of GCs

**Figure 3:**
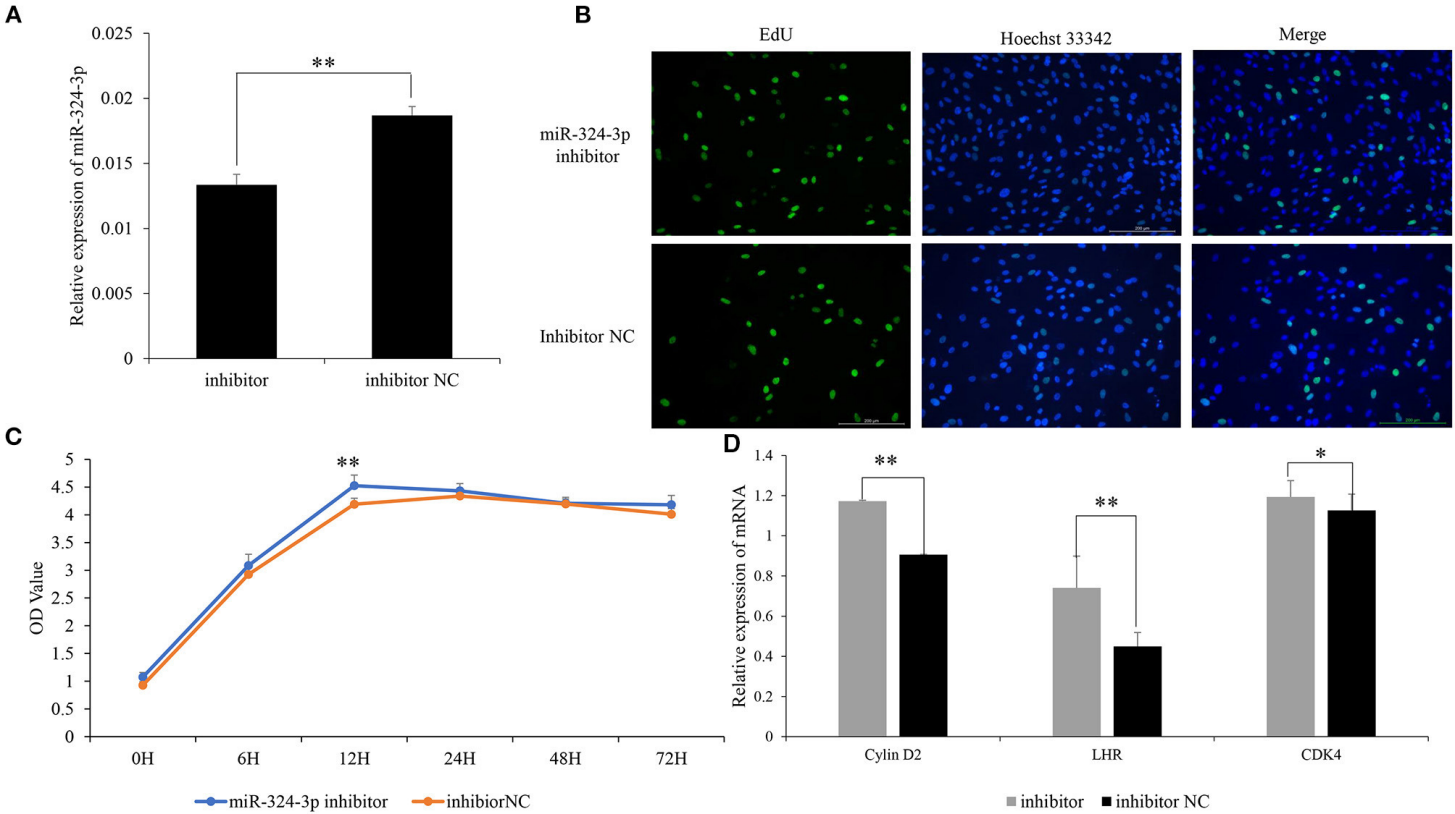
Inhibited chi-miR-324-3p expression in goat GCs. **(A)** Relative expression of miR-324-3p. **(B)** The result of EdU assay. **(C)** CCK-8 assay of goat GCs with miR-324-3p mimic and mimic NC transfection at 0–72 h. **(D)** Relative expressions of *LHR, CDK4*, and *Cylin D2* in transfected chi-miR-324-3p mimic and mimic NC at 48 h. *p < 0.05; **p < 0.01. CCK-8, Cell Counting Kit-8; GC, granulosa cell.

To further explore the role of chi-miR-324-3p in goat GCs, chi-miR-324-3p inhibitor was transfected into goat GCs (Figure 3A). The EdU and CCK-8 assay revealed that the GC proliferation in the inhibitor group was significantly higher than that of the inhibitor-NC group (Figures 3B,C) (*p* < 0.05). The mRNA expression of GC proliferation markers was significantly upregulated after inhibitor transfection (Figure 3D) (*p* < 0.05). These results demonstrated that the inhibition of chi-miR-324-3p could promote the proliferation of goat GCs.

### DENND1A Is a chi-MiR-324-3p Target

**Figure 4:**
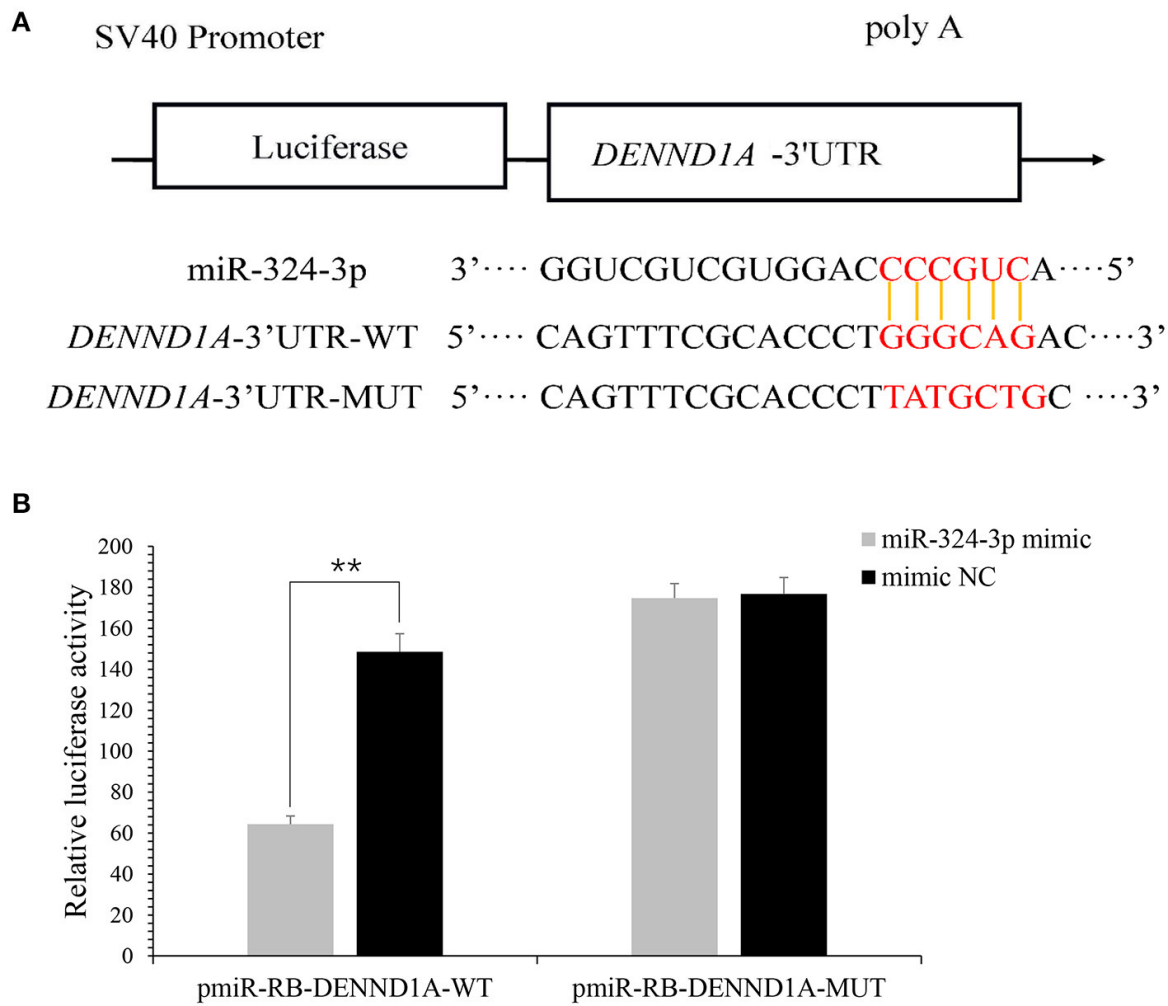
*DENND1A* is directly targeted by chi-miR-324-3p. **(A)** Predicted binding sites of chi-miR-324-3p in the 3'-UTR of *DENND1A*. The red section is the seed region of chi-miR-324-3p. **(B)** Relative luciferase activities of 293T cells cotransfected with the *DENND1A* 3'-UTR wild-type and mutant dual-luciferase reporter and chi-miR-324-3p mimic and mimic NC at 48 h. UTR, untranslated region. **P < 0.01.

In a previous study, it was revealed that *DENND1A* is a potential target of chi-miR-324-3p. To verify whether *DENND1A* is the direct target gene of chi-miR-324-3p, two plasmids containing either the WT or MUT 3'-UTR of *DENND1A* were constructed (Figure 4A). Subsequently, cotransfection of chi-miR-324-3p mimic or mimic-NC into 293T cells was performed. Notably, chi-miR-324-3p markedly decreased the activity of WT *DENND1A* compared with the negative control, but no significant changes were noted for the MUT (Figure 4B). These findings indicated that chi-miR-324-3p can directly target the 3'-UTR of *DENND1A*.

### Expression of DENND1A Is Regulated by chi-MiR-324-3p

**Figure 5:**
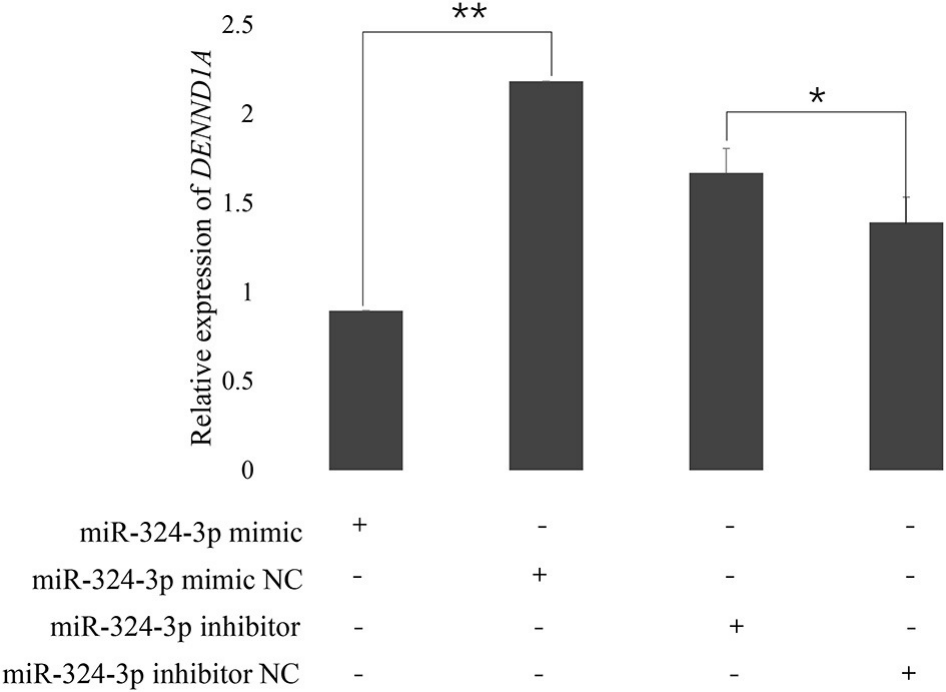
Expression of *DENND1A* in goat GCs transfected with chi-miR-324-3p mimic or mimic NC, and inhibitor or inhibitor-NC at 48 h. *RPL19* was used as the reference gene. Results are representative of the mean (±standard deviation) of three independent analyses. *p < 0.05; **p < 0.01.

To examine the validity of the putative target, chi-miR-324-3p mimic or mimic-NC was transfected into goat GCs. RT-qPCR data revealed that the transcriptional levels of *DENND1A* were different between the mimic and mimic-NC groups (Figure 5). Thus, we concluded that chi-miR-324-3p directly targets the 3'-UTR of *DENND1A* to suppress its mRNA translation in goat GCs.

## Discussion

The key candidate gene associated with prolific traits in goats remains unknown. In our study of RNA-seq, chi-miR-324-3p and *DENND1A* showed differential expression levels in high- and low-yielding goats and chi-miR-324-3p was predicted to target *DENND1A* (data unpublished). In this study, we found and validated the function of chi-miR-324-3p in goat GCs. Our results indicated that chi-miR-324-3p regulated GC proliferation by targeting *DENND1A*.

The DENN domain is a common, evolutionarily ancient, and conserved protein module; there are eight families and *DENND1A* is a member of these. *DENND1A* was detected by proteomic analysis of clathrin-coated vesicles (22). Its variant is associated with hyperandrogenism and irregular menses and is a risk variant for PCOS as documented by the NIH criteria (23). *DENND1A* is expressed in the theca cells and testes; therefore, it may be associated with hyperandrogenism by increasing androgen levels (24). In this study, the high expression of *DENND1A* was associated with prolific traits in goats; chi-miR-324-3p directly acted on the 3'-UTR of *DENND1A* in the 293T cell line. Meanwhile, our overexpression and inhibition experiments on chi-miR-324-3p further verified that the expressions of *DENND1A* and chi-miR-324-3p showed opposite trends in goat GCs. Some studies have suggested that *DENND1A* regulates Rab GTPases, which are important for calcium regulated exocytosis in pituitary cells and for basal and GnRH-induced gonadotropin release (25, 26). Moreover, *DENND1A* has been reported to participate in endocytosis and receptor trafficking in the cellular membrane. Furthermore, high methylation existed in the *DENND1A* intron in aging oocytes (16, 27). Regardless, the effects of *DENND1A* on goat GC proliferation remain experimentally unclarified. Here, we provide new insights for understanding that *DENND1A* might be a key gene involved in goat GC proliferation that is regulated by chi-miR-324-3p. Taken together, these experimental results support the conclusion that *DENND1A* is involved in GC proliferation.

A previous study reported that miR-324-3p can promote the dysregulation of the expression of genes involved in cell death and apoptosis, cAMP and Ca^2+^ signaling, cell stress, and metabolism (28). Notably, miR-324-3p inhibition was highly and significantly correlated with cell proliferation and apoptosis. miR-324-3p, which is highly upregulated in maternal blood in the second trimester, is associated with fetal growth (29). miR-324-3p has also been reported to increase the incidence of ectopic pregnancy according to the analysis of tissues obtained from voluntarily terminated pregnancy, which has a repressive interaction with 3'-UTR of *KISS1* (30). In addition, miR-324-3p overexpression in ectopic pregnancy is responsible for decreased KISS1/kisspeptin expression (31). Our previous studies have demonstrated that chi-miR-324-3p can regulate the development of ovaries in prolific goats by targeting *DENND1A* (data unpublished). Moreover, there is evidence that miR-324-3p plays crucial roles in ovary development and cell proliferation. Furthermore, miR-324-3p is related to SKOV3 cell viability, invasion, and migration *via* overexpression in ovarian cancer (32). Furthermore, a study demonstrated that miR-324-3p expression in PCOS rats is remarkably downregulated and confirmed that the overexpression of miR-324-3p represses proliferation and induces the apoptosis of GCs by targeting *WNT2B* (33). Using the CCK-8 assay, our results indicated that chi-miR-324-3p could inhibit goat GC proliferation, as demonstrated by the low expression of GC proliferation markers. We used goat ovaries in the follicular phase and the primary GCs as the animal material. Thus, we propose a new mechanism for the roles of chi-miR-324-3p in goat GCs.

In summary, our study reveals that chi-miR-324-3p regulates goat GC proliferation by directly targeting *DENND1A*. These data expand our mechanistic understanding of GC proliferation and ovary development in which miRNAs play an important role.

## Data Availability Statement

The original contributions presented in the study are included in the article/supplementary material, further inquiries can be directed to the corresponding authors.

## Ethics Statement

The animal study was reviewed and approved by the Science Research Department (in charge of animal welfare issue) of the Institute of Animal Sciences and Chinese Academy of Agricultural Sciences (IAS-CAAS) (Beijing, China) for all the experimental procedures mentioned. Ethical approval was provided by the animal ethics committee of IASCAAS (No. IAS2019-63).

## Author Contributions

Conceptualization: YFL and MXC; Methodology: YFL, ZYZ, LT and MXC; Validation: YFL, YLC and ZYZ; Formal Analysis: YFL and MXC; Investigation: YFL, ZYZ, LT, YLC, XYH, YTJ, RL, QHH and MXC; Resources: YFL, ZYZ, LT and MXC; Data Curation: YFL, ZYZ, LT, and MXC; Writing-Original Draft Preparation: YFL and MXC; Supervision: MXC; Project Administration: YFL, QHH and MXC; Funding Acquisition: QHH and MXC. All authors have read and agreed to the published version of the manuscript.

## Funding

This research was funded by the Agricultural Science and Technology Innovation Program of China (CAAS-ZDRW202106 and ASTIP-IAS13), the China Agriculture Research System of MOF and MARA (CARS-38), the Major Science and Technology Special Plan of Yunnan Province (202102AE090039), and the Basic Research Foundation Key Project of Yunnan Province (202001AS070002).

## Conflict of Interest

The authors declare that the research was conducted in the absence of any commercial or financial relationships that could be construed as a potential conflict of interest.

## Publisher's Note

All claims expressed in this article are solely those of the authors and do not necessarily represent those of their affiliated organizations, or those of the publisher, the editors and the reviewers. Any product that may be evaluated in this article, or claim that may be made by its manufacturer, is not guaranteed or endorsed by the publisher.
